# Auricular acupuncture for insomnia in female patients with non-metastatic breast cancer: study protocol for a multicenter, randomized, controlled clinical trial

**DOI:** 10.3389/fpsyt.2026.1816187

**Published:** 2026-05-25

**Authors:** Lumin Liu, Shihan Jiang, Meina Ye, Yue Zhang, Zhicheng Guo, Yingying Hu, Xi Wang, Yuelai Chen, Yiting Jin, Wei Zhu, Ping Yin

**Affiliations:** 1 Sleep Medicine Centre, LongHua Hospital Shanghai University of Traditional Chinese Medicine, Shanghai, China; 2 Department of Breast Surgery, LongHua Hospital Shanghai University of Traditional Chinese Medicine, Shanghai, China; 3 Department of General Surgery, Huashan Hospital, Fudan University, Shanghai, China; 4 Department of General Surgery, Zhongshan Hospital, Fudan University, Shanghai, China

**Keywords:** auricular acupuncture (AA), breast cancer, female, insomnia, non-metastatic

## Abstract

**Background:**

Breast cancer patients are at an increased risk of insomnia, which can lead to emotional disorders, reduced quality of life, and potentially higher cancer mortality. Auricular acupuncture (AA) offers a feasible treatment option due to its short procedure time, low technical complexity, and relative safety. However, while preliminary studies suggest potential benefits of AA for treating insomnia, the evidence for the population of breast cancer patients remains insufficient.

**Methods:**

This study employs a multicenter single-blind, randomized, controlled trial. A total of 324 patients will be randomly assigned in a 1:1 ratio to either the AA group (n=162) or the nonspecific AA group (NAA) (n=162). Both groups will undergo a 4-week treatment period, with three daily sessions. Follow-up assessments will be conducted at weeks 8 and 16. The primary outcome is the treatment response rate at week 4, and secondary outcomes include the treatment remission rate, the treatment response rate at weeks 8 and 16, change in Insomnia Severity Index (ISI) score, sleep diary, Generalized Anxiety Disorder-7 (GAD-7), Patient Health Questionnaire-9 (PHQ-9), Quality of Life Questionnaire-Core 30 (QLQ-C30), and emergency medication usage. Data will be collected at baseline (week 0), during the intervention (week 2), post-treatment (week 4), and at follow-up (weeks 8 and 16).

**Discussion:**

This study represents an initial effort to develop a comprehensive standard operating procedure (SOP) that includes graphic and video instructions for auricular point location and manipulation techniques. It also provides online guidance to instruct patients on how to self-administer AA at home. The aim of the study is to evaluate the clinical efficacy and safety of AA in treating insomnia among female patients with non-metastatic breast cancer. The findings will provide new insights into the promotion of AA for managing breast cancer-related insomnia in a home-use setting, a method that has the potential to conserve medical resources and alleviate economic burdens.

**Trial registration:**

https://itmctr.ccebtcm.org.cn/mgt/project/view/-7546531521559384755, identifier ITMCTR2025001101.

## Background

Breast cancer is the most prevalent cancer among women worldwide, accounting for 11.6% of all cancer cases and serving as a leading cause of cancer-related deaths (1). According to the 2022 Global Cancer Statistics, approximately 2.3 million new cases and 665,000 deaths occur annually (1). Insomnia is a prevalent clinical issue among cancer patients, occurring at a rate approximately three times higher than in the general population (2). It can manifest at any stage of cancer and is particularly common in breast cancer patients (3, 4). Insomnia not only heightens the risk of mental and physical comorbidities but also impairs patient adherence to cancer treatments, thereby negatively impacting treatment outcomes (5). Furthermore, sleep quality significantly influences immune function, neuroendocrine function, cognitive health, and overall quality of life (6).

Current interventions for insomnia in breast cancer patients primarily include pharmacotherapy, cognitive behavioral therapy, acupuncture, and Tai Chi. Although numerous studies have demonstrated the effectiveness of these interventions in improving sleep in breast cancer patients, limitations persist (7–9). As such, effective and proactive management of insomnia in this patient population remains a critical challenge in clinical practice.

AA, a non-pharmacological approach, is based on the principle that the auricle corresponds to the body’s internal organs. Specific regions of the auricle are innervated by the auricular branch of the vagus nerve, which is the only cutaneous branch of this nerve and plays a crucial role in regulating various physiological processes in humans (10). By stimulating specific auricular points, the auricular branch of the vagus nerve is activated, which in turn regulates the function it innervates and treats diseases like insomnia (11, 12). In this trial, “auricular acupuncture” refers to the insertion and retention of intradermal needles at specified auricular points, combined with patient self-administered acupressure and home-based needle replacement following the video SOP and operation manual. While preliminary studies have suggested the clinical efficacy of AA for insomnia, limitations remain, particularly among breast cancer patients (13, 14). Existing studies are primarily single-center, small-sample investigations, and there is a lack of well-designed control groups, leading to considerable heterogeneity in the evaluation of treatment outcomes (15).

To address these gaps, we have designed a multicenter, randomized controlled trial to assess the clinical efficacy and safety of AA, compared to NAA, in treating insomnia in breast cancer patients. This study aims to provide robust, evidence-based findings to support the use of AA for female patients with non-metastatic breast cancer, thereby informing clinical treatment strategies.

## Methods

### Study design

**Figure 1:**
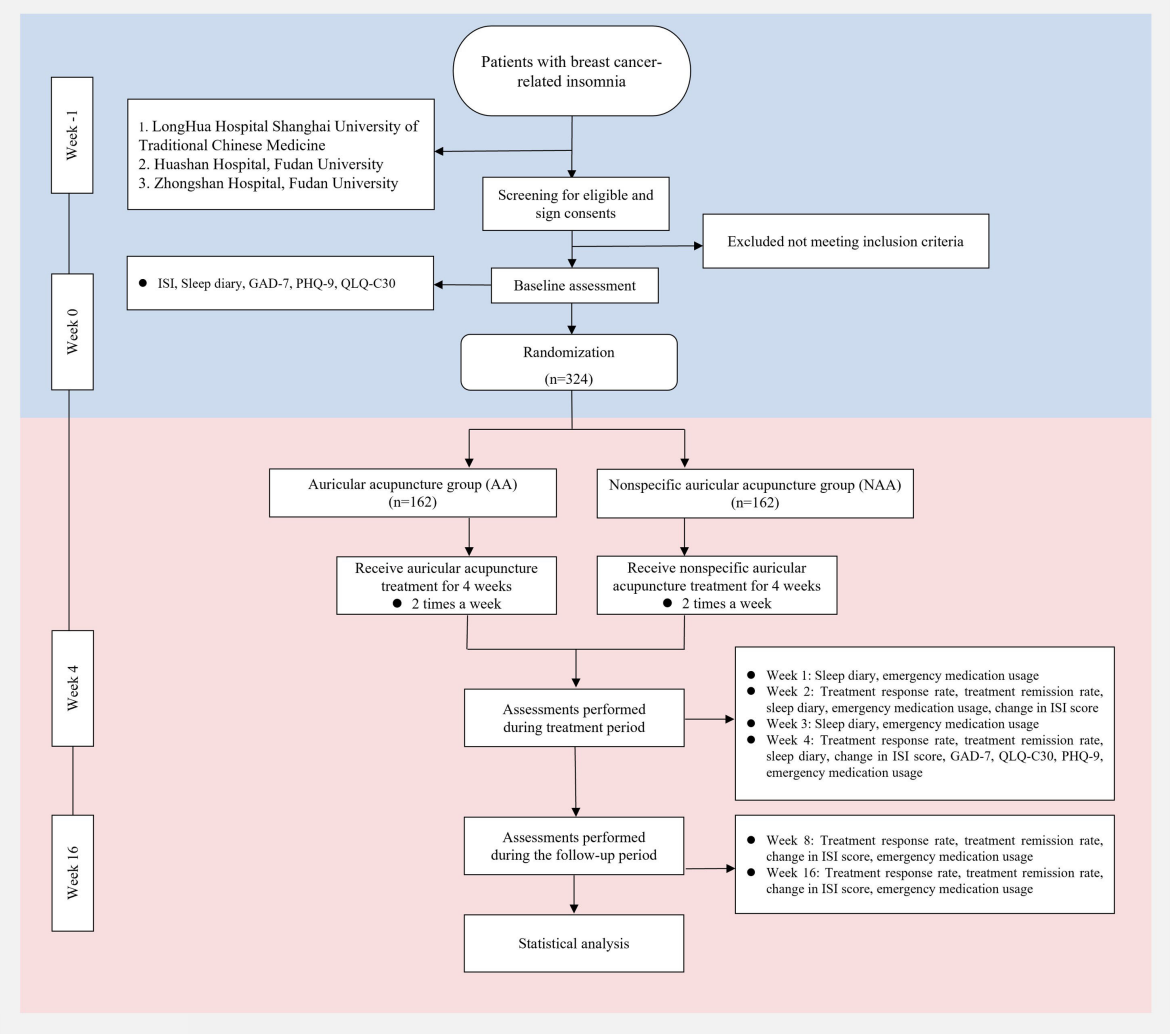
Trial flow chart. ISI, Insomnia Severity Index; GAD-7, Generalized Anxiety Disorder Scale-7; PHQ-9, Patient HealthQuestionnaire-9; QLQ-C30, Quality of Life Core Scale-30.

This study will be conducted across three centers, including LongHua Hospital, Shanghai University of Traditional Chinese Medicine; Zhongshan Hospital, Fudan University; and Huashan Hospital, Fudan University. A total of 324 patients will be recruited and randomly assigned in a 1:1 ratio to either the AA group or the NAA group. Both groups will undergo a 4-week treatment, followed by a 12-week follow-up. **Figure 1** illustrates the flowchart of the trial, and **Table 1** presents a schematic timeline outlining the recruitment process, intervention schedule, and efficacy evaluations. The protocol for this study was based on the Standard Protocol Items: Recommendations for Intervention Trials (SPIRIT) 2013, as outlined in **Supplementary Material 1**.

**Table 1:** Schedule of enrolment, interventions and assessment times.

Study period	Enrolment		Intervention period				Follow-up period	
Time point	Week -1	Week 0	Week 1	Week 2	Week 3	Week 4	Week 8	Week 16
Eligibility screen	**×**							
Sign informed consent	**×**							
randomization		**×**						
Interventions								
Auricular acupuncture								
Nonspecific auricular acupuncture								
Primary outcomes								
Treatment response rate						**×**		
Secondary outcomes								
Treatment remission rate				**×**		**×**	**×**	**×**
Treatment response rate				**×**			**×**	**×**
Change in ISI score				**×**		**×**	**×**	**×**
Sleep diary								
GAD-7		**×**				**×**		
PHQ-9		**×**				**×**		
QLQ-C30		**×**				**×**		
Adverse events			**×**	**×**	**×**	**×**		
Emergency medication usage			**×**	**×**	**×**	**×**	**×**	**×**
Success of blinding						**×**		

### Patients

Recruitment will be conducted through the hospital’s official online platform and posters. Patients who meet the inclusion criteria will be invited for a face-to-face interview and baseline assessment. Before signing the informed consent form (ICF), all patients will be thoroughly briefed on the potential benefits, risks, and study requirements through both verbal explanations and written materials. Eligible patients will then be randomly assigned to either the AA or the NAA group. Patients may withdraw from the study at any time.

### Inclusion criteria

(1) Participants are required to have stage I-III breast cancer, diagnosed and staged according to the NCCN Clinical Practice Guidelines (2022, 2nd ed.) and the AJCC Cancer Staging Manual (8th ed.), respectively. Insomnia will be assessed with reference to Diagnostic and Statistical Manual of Mental Disorders, 5th Edition (DSM-5), with symptoms occurring at least three nights per week for ≥1 month after breast cancer diagnosis and related to the malignancy or its treatment. (2) Female patients aged between 18 and 75 years. (3) Eastern Cooperative Oncology Group (ECOG) performance status score of ≤2. (4) ISI score of ≥8. (5) An estimated survival period of ≥6 months. (6) Written ICF to participate in the study.

### Exclusion criteria

(1) Those with severe heart, liver, kidney or other major diseases; (2) Those who are pregnant, lactating or planning to become pregnant within the next month; (3) Those with surgical plans during the trial period; (4) Those with a history of drug abuse or addiction; (5) Those who have taken sedative-hypnotic or antipsychotic drugs within two weeks before baseline, or have received other insomnia treatments (such as cognitive behavioral therapy, etc.) within three months before baseline; (6) Those with insomnia caused by cancer pain (NRS score≥4) or other physical diseases; (7) Those with mental or intellectual abnormalities who cannot finish the assessment; (8) Those who have long-term night work or irregular schedules; (9) Those who have received AA treatment within three months prior to enrollment; (10) Those with ulcers, abscesses, skin infections or other conditions at the treatment sites; (11) Those allergic to metals and adhesive tapes; (12) Those with ear deformities, perforations, cartilage deformations or other conditions; (13) Those with auricular cartilage inflammation or other serious complications related to the ear; (14) Those who have participated in other clinical medical trials within the past month.

### Randomization, allocation concealment and blinding

The random allocation scheme for this study will be generated by researchers at the Clinical Research Center of LongHua Hospital, Shanghai University of Traditional Chinese Medicine, who will not be involved in participant recruitment, intervention delivery, outcome assessment, or statistical analysis. A block randomization method will be employed, with allocation codes in opaque envelopes provided by the data management center. Patients will be informed that they have an equal chance of being assigned to either the AA group or the NAA group. Moreover, strict adherence to the principle of departmental separation will be enforced to ensure the reliability and validity of the research.

A single-blind research design will be adopted, whereby all patients, evaluators, and statistical analysts, except for the acupuncturists, will remain unaware of group allocations. To facilitate successful blinding, all researchers will undergo rigorous training on the standardized implementation of the research protocol for two groups, including standardized communication, similar external appearance of the adhesive devices and procedural instructions, separation of appointment times to reduce communication between participants, restriction of communication regarding group allocation, and separation of acupuncturists from outcome assessment and data analysis. Outcome assessors will remain blinded throughout the trial and will not participate in intervention delivery. After the 4-week intervention, participants will be asked to guess group allocation as “AA”, “NAA” or “uncertain” and blinding integrity will be evaluated using the Bang Blinding Index. Unblinding will be permitted only when required for participant safety or trial management, and all unblinding events will be documented.

### Intervention

Throughout the study, all patients will receive the same standardized sleep hygiene education, which includes guidance on avoiding caffeine, tea, alcohol, and other stimulating beverages, maintaining regular daily schedules and appropriate sleep environments, and managing emotional well-being. Patients will be required to maintain a sleep diary for five weeks, documenting their sleep patterns at the following time points: week 0 - week 4.

All acupuncturists from the three centers will receive centralized standardized training before trial initiation, covering auricular point location, device application, insertion depth, disinfection procedures, patient instruction, and adverse event identification. Regular cross-center quality control meetings will be conducted to ensure protocol consistency.

Before home-based self-administration, participants will receive standardized training from trained acupuncturists, including face-to-face demonstration, video SOP instruction, and written operation manuals (**Supplementary Material 2**). During the first and fifth treatments, acupuncturists will verify participants’ ability to correctly locate auricular points, disinfect the auricle, apply the device, and perform pressing stimulation. During home treatment, participants will record device replacement and daily stimulation, upload operation photographs or videos weekly for researcher verification, and complete electronic adherence check-ins through WeChat. If incorrect operation or poor adherence is identified, researchers will provide timely guidance and retraining. A contact number will be provided for reporting adverse events (AEs) or operational problems.

If a patient experiences severe insomnia-related discomfort for two consecutive days or more, emergency medication usage may be used based on clinical need. The use of rescue medication is intended for short-term symptomatic relief and is not encouraged as routine treatment. Researchers will carefully record the name, dosage, frequency, and duration of medication use in the Case Report Form (CRF).

### Auricular acupuncture group

**Figure 2:**
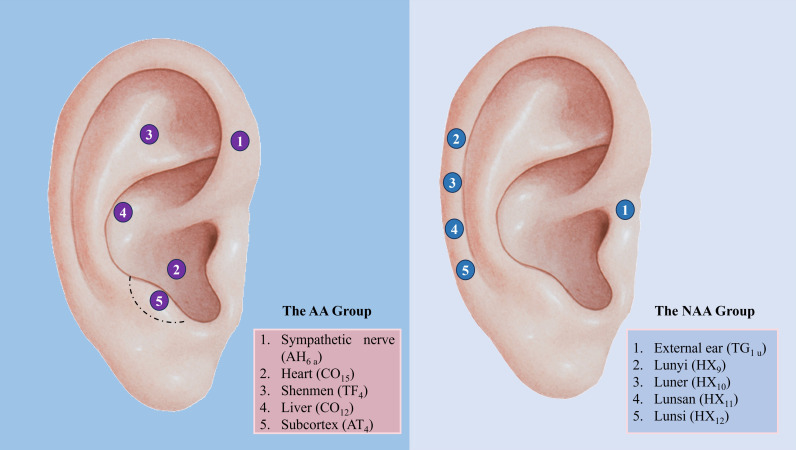
The locations of auricular acupoints in both the AA and NAA groups.

**Figure 3:**
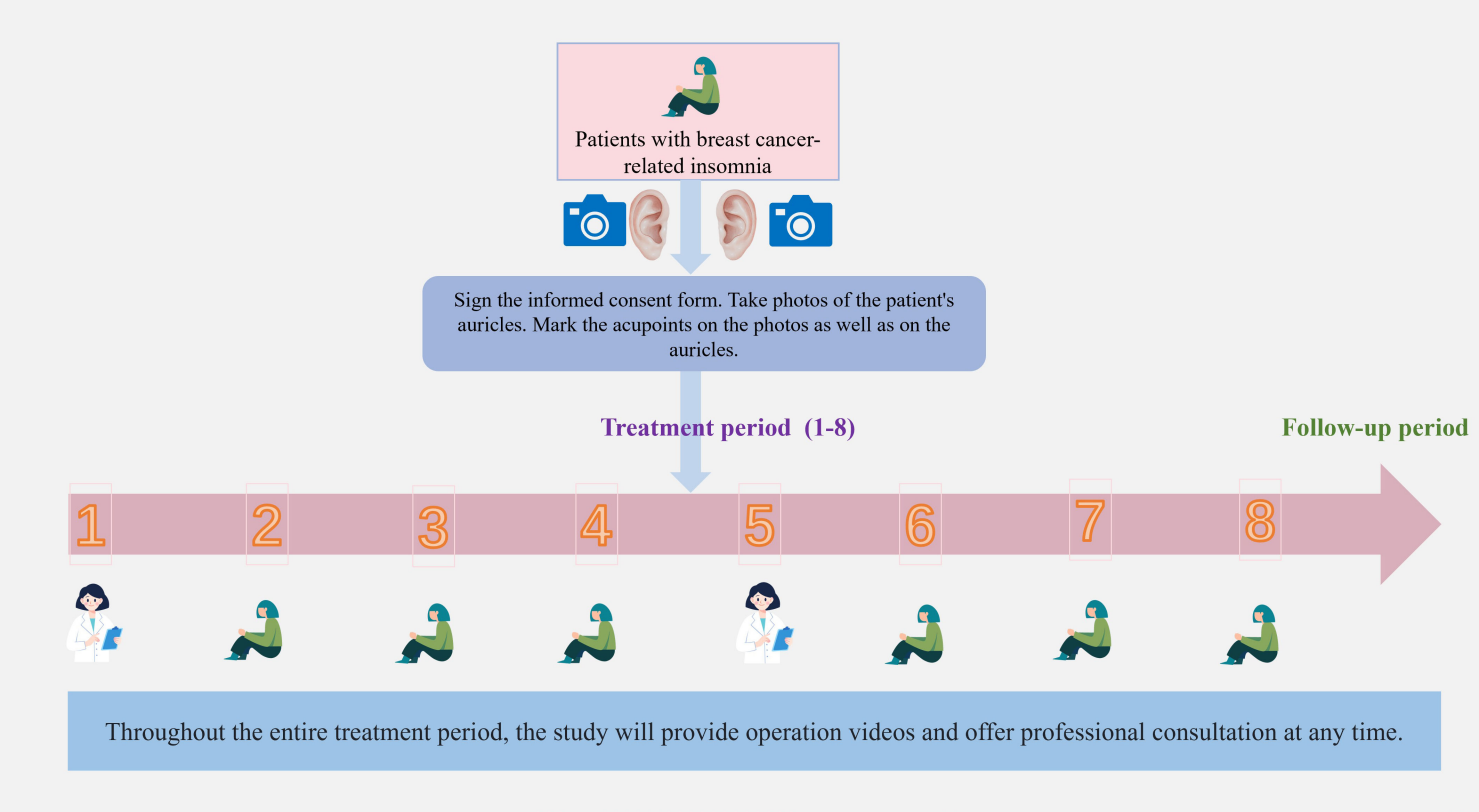
Treatment flow chart.

Patients in AA group will receive treatment at five auricular acupoints: subcortex (AT_4_), heart (CO_15_), shenmen (TF_4_), liver (CO_12_), and sympathetic nerve (AH_6a_) (11) (**Figure 2**; **Table 2**). Sterile AA devices (0.20*1.5mm) from SEIRIN CORPORATION (Japan) will be used. Prior to treatment, the auricular acupoints will be disinfected using 75% alcohol. The acupuncture devices will be inserted to a depth of 1.5mm and secured with adhesive tape. Each acupoint will be manually stimulated by finger pressure for 30 seconds or until the auricle turns red, with stimulation occurring three times daily (11, 16). The AA devices will be replaced twice a week (every three days), alternating between the left and right auricular acupoints. This process will entail a total of eight applications over a 4-week period, followed by a 12-week follow-up (**Figure 3**). The first and fifth treatments will be administered by a trained acupuncturist.

**Table 2:** The locations of auricular acupoints in both the AA and NAA groups.

Group	Auricular acupoint	Location
Auricular acupuncture group (AA)	Sympathetic nerve (AH_6 a_)	At the junction of the anterior end of the inferior crus of the antihelix and the inner margin of the helix, which corresponds to the anterior end of the sixth region of the antihelix.
	Heart (CO_15_)	In the central depression of the concha, specifically in the 15th area of the concha.
	Shenmen (TF_4_)	In the upper part of the posterior one-third of the triangular fossa, specifically in area 4 of the triangular fossa.
	Liver (CO_12_)	In the posterior inferior part of the concha, specifically in the 12th region of the concha.
	Subcortex (AT_4_)	On the medial side of the antitragus, specifically in the 4th region of the antitragus.
Nonspecific auricular acupuncture group (NAA)	External ear (TG_1 u_)	In front of the incisura on the screen, near the helix, that is, at the upper edge of the tragus area 1.
	Lunyi (HX_9_)	At the helix below the helix tubercle, which is helix region 9.
	Luner (HX_10_)	At the helix below wheel 1, which is helix region 10.
	Lunsan (HX_11_)	At the helix below wheel 2, which is helix region 11.
	Lunsi (HX_12_)	At the helix below wheel 3, which is helix region 12.

### Nonspecific auricular acupuncture group

In the NAA group, External ear (TG_1u_), Lunyi (HX_9_), Luner (HX_10_), Lunsan (HX_11_), Lunsi (HX_12_) will be used (11). Sterile AA devices (specifications: 0.20*0.3mm) from SEIRIN CORPORATION (Japan) will be employed, with needles being inserted to a depth of 0.3mm. It is important to note that the retention time and replacement method for the AA devices in the NAA group will follow the same protocol as that of the AA group.

### Outcomes

#### Primary outcome

The primary outcome is the treatment response rate, defined as the percentage of patients whose total ISI score decreases by ≥8 points compared to the baseline score at the end of the 4-week treatment period (17, 18).

The ISI is a reliable and valid self-report tool used to measure insomnia. It assesses aspects such as difficulty falling asleep, staying asleep, waking up early, sleep satisfaction, daytime impairment, and overall quality of life. The ISI uses a 7-item scoring system, with total scores ranging from 0 to 28, where higher scores indicate more severe insomnia. According to clinical criteria, scores of 0–7 indicate no significant insomnia, 8–14 indicate subthreshold insomnia, 15–21 indicate moderate insomnia, and 22–28 indicate severe insomnia.

#### Secondary outcome

The secondary outcomes include the following eight indicators:

1. Treatment remission rate: The percentage of patients achieving an ISI score <8 at weeks 2, 4, 8, and 16.
2. Treatment response rate at weeks 2, 8, and 16.
3. Sleep diary (19): Total sleep time (TST), sleep onset latency (SOL), number of sleep awakenings (SA), wake after sleep onset (WASO), early morning awakenings (EMA), average duration of awakenings (ADA), and sleep efficiency (SE), as recorded in sleep diaries at weeks 0, 1, 2, 3, and 4.
4. Change in ISI score: This will be evaluated at weeks 2, 4, 8, and 16.
5. Generalized Anxiety Disorder-7 (GAD-7) (20): This scale assesses anxiety symptoms experienced by patients in the past two weeks. Evaluations will be conducted at Weeks 0 and 4.
6. Patient Health Questionnaire-9 (PHQ-9) (21): This scale evaluates depression symptoms in the past two weeks. Evaluations will take place at Weeks 0 and 4.
7. Quality of Life Questionnaire-Core 30 (QLQ-C30) (22): This 30-item scale assesses the quality of life among cancer patients across six dimensions and is widely used in clinical research. Assessment will be conducted at Weeks 0 and 4.
8. Emergency medication usage: The percentage of patients using emergency medication usage, as well as the dosage, frequency, and duration of medication use, will be recorded at weeks 1, 2, 3, 4, 8, and 16.

#### Adverse events

AEs associated with AA may include redness at the needle site, ear swelling, fainting due to the needle, local infections, and exacerbation of symptoms due to excessive stimulation. The severity of these AEs and causality between AEs and the intervention will be assessed by researchers and recorded in detail in the CRF. In the event of serious AEs, researchers will promptly report them to the ethics committee and will determine whether to suspend or withdraw the trial. The incidence of AEs will be calculated as the percentage of patients experiencing AEs during the trial. The intervention may be discontinued if participants request withdrawal, experience serious adverse events, or are judged unsuitable for continued treatment by investigators.

### Data collection, management and monitoring

Data collection and entry will be conducted by two independent researchers who are not involved in randomization, treatment, or evaluation. To ensure consistency, both researchers will independently enter the data and perform data validation, thereby ensuring the accuracy and completeness of the data. Participant data will be de-identified and access to identifiable information will be restricted to authorized study personnel only. Additionally, all research data will be reviewed by the Data Security Management Committee to monitor trial compliance and safety.

### Statistical methods

#### Sample size

The treatment response rate at week 4 serves as the primary outcome (23). A bilateral significance level (α) of 0.05 and a statistical power (1-β) of 0.9 were established. Based on our unpublished pilot data, in which the study design, intervention protocol, control condition, and outcome definition were highly consistent with the present trial, the treatment response rate for AA group at week 4 is anticipated to be 40.00%, while the NAA group is 22.22%. Using PASS 15.0 software, the required sample size was calculated to be N1 = N2 = 137 for both groups. Accounting for a 15% dropout rate, a total of 162 patients will be recruited in each group, resulting in an overall total of 324 patients.

### Statistical analysis

This study will utilize three databases: the Full Analysis Set, the Per-Protocol Set, and the Safety Set. Statistical analyses will be performed using SAS 9.4 software (SAS Institute Inc., Cary, North Carolina, USA). Continuous variables will be reported as mean and standard deviation or median (interquartile range), while categorical variables will be expressed as frequency (proportional composition). A *P*-value <0.05 will be considered statistically significant.

The primary outcome, the treatment response rate at week 4, will be compared using the Cochran-Mantel-Haenszel test. Secondary outcomes, including treatment response rate, treatment remission rate, and emergency medication usage, will be analyzed as qualitative data with repeated measures using generalized estimating equations. Changes in TST, SOL, SA, WASO, EMA, ADA, and SE as well as changes in ISI, GAD-7, PHQ-9, and QLQ-C30 scores, will be analyzed as quantitative data using mixed-effects models. Secondary outcomes will be considered supportive and exploratory, thus no formal adjustment for multiple comparisons will be applied. The incidence of adverse events will be analyzed using chi-square tests or Fisher’s exact tests. Missing data will be handled using mixed-effects models under the missing-at-random assumption. Multiple imputation will be performed as a sensitivity analysis to assess the robustness of the primary results. Key clinical and treatment-related variables, including study center, TNM stage, cancer treatment modalities, pain level, menopausal status, baseline ISI severity, and emergency medication usage, will be considered as pre-specified covariates where appropriate.

## Discussion

The incidence of insomnia related to breast cancer is notably high, significantly impacting patients’ quality of life and prognosis, thereby constituting a pressing public health concern. Current pharmacological treatments primarily involve sedative-hypnotics; however, these medications are associated with addictive properties, drug resistance, rebound symptoms upon discontinuation, and adverse daytime effects, which restrict their clinical applicability.

In exploring non-pharmacological interventions for insomnia in breast cancer patients, acupuncture has emerged as a recognized therapeutic option (8, 24). Nevertheless, the time and financial costs can be relatively high, leading to suboptimal patient compliance and often failing to achieve the desired therapeutic effects under rigorous RCT conditions (25). In addition, many patients with breast cancer experience physical limitations, treatment-related fatigue, frequent hospital visits, and travel burden, which may reduce access to repeated in-hospital non-pharmacological treatments. Therefore, a safe, low-cost, and standardized home-based intervention may be clinically meaningful for this population. Consequently, this study focuses on AA as a potential solution to these challenges.

Previous research has established the efficacy of AA in alleviating depression, with insomnia being a prevalent clinical symptom of this condition (11). Investigations have also demonstrated the effectiveness of AA in treating primary insomnia (26). Unlike pharmacological treatments, AA does not exhibit addictive or drug-resistant properties, and any adverse events are typically transient, mild, and manageable (11). Furthermore, AA is more feasible for implementation due to its shorter manipulation time, lower technical complexity, and relative safety. It can also be performed at home following a standardized SOP. It is particularly suitable for postoperative individuals with compromised physical strength, limited mobility, or those undergoing frequent chemotherapy, thereby enhancing patient compliance. Additionally, AA incurs lower economic costs, alleviating the financial burden on cancer patients and the broader healthcare system.

This study aims to rigorously evaluate the clinical efficacy and safety of AA in treating insomnia among female patients with non-metastatic breast cancer. To reduce clinical heterogeneity, this study includes only female patients with non-metastatic breast cancer and excluded patients with insomnia primarily caused by moderate-to-severe cancer pain. Male breast cancer is rare and may differ from female breast cancer in biological, hormonal, and psychosocial characteristics. Pain-dominant insomnia may require priority analgesic treatment and involve different mechanisms, which could confound evaluation of the effect of AA on insomnia.

A comprehensive SOP will be developed to guide patients in self-administering the treatment, incorporating both graphic and video instructions for auricular point locations and operational techniques, alongside online support to ensure treatment standardization and adherence. The home-based self-administered AA model is a major feature of this trial. The use of written manuals, video SOPs, online guidance, operation verification, and adherence monitoring may improve accessibility and standardization. However, this model also introduces challenges related to point location, insertion accuracy, stimulation intensity, adherence, and adverse event monitoring.

Regarding blinding, existing literature acknowledges the challenges in establishing control protocols due to the high sensitivity and innervation of the auricular pavilion, which renders nonspecific needling physiologically active (27). Therefore, the NAA group was designed as a minimal active control with nonspecific auricular stimulation rather than a completely inert placebo. To minimize specific therapeutic effects, nonspecific auricular points not known to be associated with sleep regulation were selected (28), and a shallow insertion depth of 0.3 mm was used. These design elements are intended to contribute to the generation of high-quality, evidence-based medical findings.

However, the current study design does have limitations. First, the inclusion of only female patients with non-metastatic breast cancer and the exclusion of patients with moderate-to-severe cancer pain or advanced/metastatic disease may limit generalizability. Second, although the NAA protocol was designed to minimize specific therapeutic effects, differences in point selection, insertion depth, and device specifications may influence the interpretation of between-group differences. Third, reliance on self-reported sleep outcomes may introduce expectancy and placebo effects. Future studies should consider objective sleep assessments, such as actigraphy, when feasible. In conclusion, we anticipate that the findings of this study will optimize AA treatment for breast cancer patients, ultimately enhancing their quality of life.

## Trial status

Patients’ recruitment is anticipated to commence on 01 March 2026 and is expected to be completed by 01 March 2028.

## Dissemination

The trial results will be disseminated through peer-reviewed publication.
