# Supplementary material for: Auricular acupuncture for insomnia in female patients with non-metastatic breast cancer: study protocol for a multicenter, randomized, controlled clinical trial
Source: Front Psychiatry. 2026 May 25;17:1816187. doi: 10.3389/fpsyt.2026.1816187 (PMC13243390; doi:10.3389/fpsyt.2026.1816187)
Supplement: Supplementary file 1 [file Supplementaryfile1.docx]

**Auricular Acupuncture Operation Guide**

**(Patient Self-Use Version)**

(Please use under the guidance of a doctor to ensure correct acupoint selection)

**1. Preparation of Supplies**

**• Auricular Acupuncture:** Disposable sterile auricular acupuncture (provided by researchers).

**• Disinfection Supplies**: 75% alcohol cotton balls or iodine swabs (for those allergic to alcohol), sterile cotton balls or swabs.

**• Auxiliary Tools**: A mirror (or with family assistance, refer to your individual auricular acupoint photos and pre-labeled acupoints marked by the doctor on the auricle to locate corresponding acupoints sequentially).

**2. Environmental Requirements**

The operation environment should be clean, quiet, well-lit, and at a comfortable temperature.

**3. Cleaning and Disinfection**

**• Handwashing**: First, wash your hands thoroughly with running water and soap for at least 20 seconds, then dry with a clean towel or tissue.

**• Auricle Cleaning**: Gently wipe the auricle with sterile cotton balls or swabs to remove oil and debris, ensuring the area is clean.

**• Skin Disinfection**: Using 75% alcohol cotton balls (or iodine swabs), disinfect the skin in a spiral motion from the center of the corresponding auricular acupoint outward. Repeat 2–3 times per acupoint and allow several seconds to dry.

**4. Auricular Acupuncture Application**

① Open the needle package and use disposable sterile tweezers (or clean, disinfected hands) to hold the back of the needle patch. Align the needle tip with the acupoint and apply it accurately to the selected auricular acupoint.

② Press the needle’s handle with your finger to secure it to the skin, then press and stimulate the corresponding acupoint area (3–5 seconds per acupoint) to ensure the patch adheres firmly.

③ Press each acupoint for 30 seconds or until the auricle slightly reddens. Stimulate each acupoint 3 times daily. Researchers will remind patients to complete acupoint pressing via online platforms, phone calls, or WeChat daily.

④ Each auricular acupuncture can be retained for 3 days. On the 4th day, remove it and switch to the opposite auricle for treatment, alternating between both auricles in this cycle. Mild tingling or soreness in the ear during this period is normal.

**Note:** This study involves a total of 4 weeks of treatment. Throughout the study, auricular acupuncture needs to be replaced twice weekly (alternating between left and right ears), totaling 8 treatments. Except for the 1st and 5th treatments performed by researchers, you will complete all other replacements independently. (Researchers will provide relevant demonstration videos for these steps.)

**5. Daily Care**

During auricular acupuncture treatment, keep the ear skin clean and dry to prevent infection. Although the ear auricular acupunctures used in this study have water-resistant properties and allow normal daily activities like washing and bathing, avoid prolonged immersion in water. If an auricular acupuncture falls off, replace it using the method described in "**4. Auricular Acupuncture Application**."

**6. Needle Removal**

Use tweezers to lift a corner of the adhesive patch and gently peel it off along the skin. If adhesive residue remains, gently wipe it clean with a 75% alcohol cotton ball (or iodine swab).

After removing the needle, mild pain, redness, or minor bleeding at the site is normal and will generally resolve on its own. If abnormal conditions such as local redness, increased pain, or fever occur, seek medical attention promptly.

**7. Observation and Management**

① During treatment or needle retention, if you experience syncope-like symptoms (dizziness, palpitations, sweating), stop the operation immediately and remove the needle. Lie flat, loosen tight clothing, keep warm, and drink warm water or sugary water. Seek emergency care if necessary.

② Monitor the ear skin for redness, swelling, itching, or exudate. For mild discomfort, adjust the pressing force on the needle; for severe issues like infection, remove the needle immediately, disinfect the area, and seek appropriate medical treatment based on your condition.

**8. Precautions**

**Do not perform self-treatment**: If you have ulcers, boils, skin infections at the acupoint site, or are allergic to metals or adhesive tape. Consult researchers first.

**Do not reuse auricular acupunctures**: All needles in this study are single-use sterile devices. Dispose of used needles in a lidded container—do not mix with household waste.

**Adhere to the treatment schedule**: Ear acupoint therapy requires regular application. If you miss a session, apply the needle as soon as possible. Additionally, do not retain needles for longer than 3 days.

If you have any questions during the study, contact our research team at any time!

Wishing you a speedy recovery!
